# Curcumin and Radiotherapy Exert Synergistic Anti-Glioma Effect In Vitro

**DOI:** 10.3390/biomedicines9111562

**Published:** 2021-10-28

**Authors:** Vasiliki Zoi, Vasiliki Galani, Evrysthenis Vartholomatos, Natalia Zacharopoulou, Eftichia Tsoumeleka, Georgios Gkizas, Georgios Bozios, Pericles Tsekeris, Ieremias Chousidis, Ioannis Leonardos, Andreas G. Tzakos, Athanasios P. Kyritsis, George A. Alexiou

**Affiliations:** 1 Neurosurgical Institute, University of Ioannina, 45500 Ioannina, Greece; vasozoi95@gmail.com (V.Z.); eyrys.varth@gmail.com (E.V.); nataliezacharop13@gmail.com (N.Z.); eutuxiatsoum@gmail.com (E.T.); geogkizas@hotmail.com (G.G.); thkyrits@uoi.gr (A.P.K.); 2 Department of Anatomy Histology-Embryology, School of Medicine, University of Ioannina, 45110 Ioannina, Greece; vgalani@uoi.gr; 3 Department of Medical Physics, University of Ioannina, 45110 Ioannina, Greece; gbozios@uhi.gr; 4 Department of Radiation Oncology, University of Ioannina, 45110 Ioannina, Greece; ptsekeri@uoi.gr; 5 Zoology Laboratory, Department of Biological Application and Technology, University of Ioannina, 45110 Ioannina, Greece; i.chousidis@uoi.gr (I.C.); ileonard@uoi.gr (I.L.); 6 Department of Chemistry, Section of Organic Chemistry and Biochemistry, University of Ioannina, 45110 Ioannina, Greece; atzakos@uoi.gr

**Keywords:** curcumin, glioblastoma cells, radiation

## Abstract

Curcumin, a bioactive polyphenol, is known to have anticancer properties. In this study, the effectiveness of curcumin pretreatment as a strategy for radio-sensitizing glioblastoma cell lines was explored. For this, U87 and T98 cells were treated with curcumin, exposed to 2 Gy or 4 Gy of irradiation, and the combined effect was compared to the antiproliferative effect of each agent when given individually. Cell viability and proliferation were evaluated with the trypan blue exclusion assay and the 3-(4,5-dimethylthiazol-2-yl)-2,5-diphenyltetrazolium bromide (MTT) assay. The synergistic effects of the combination treatment were analyzed with CompuSyn software. To examine how the co-treatment affected different phases of cell-cycle progression, a cell-cycle analysis via flow cytometry was performed. Treatment with curcumin and radiation significantly reduced cell viability in both U87 and T98 cell lines. The combination treatment arrested both cell lines at the G2/M phase to a higher extent than radiation or curcumin treatment alone. The synergistic effect of curcumin when combined with temozolomide resulted in increased tumor cell death. Our results demonstrate for the first time that low doses of curcumin and irradiation exhibit a strong synergistic anti-proliferative effect on glioblastoma cells in vitro. Therefore, this combination may represent an innovative and promising strategy for the treatment of glioblastoma, and further studies are needed to fully understand the molecular mechanism underlying this effect.

## 1. Introduction

Glioblastoma is the most common and severe Central Nervous System (CNS) tumor accounting for 45.6% of all primary malignant brain tumors [1,2]. In spite of intensive clinical investigation, the median survival remains around 15 months, and recurrence is almost universal since the tumor shows significant resistance to all existing therapeutic approaches. Many chemotherapeutic agents have been used against glioblastoma, including temozolomide (TMZ) [3,4]. Chemoresistance has been proven to be a major challenge for successful treatment, and several different chemoresistance mechanisms have been investigated and reported [5]. The presence of the blood–brain barrier and the highly aggressive infiltration of glioblastoma into the surrounding tissues has, so far, surmounted effective treatment [6].

**Figure 1:**
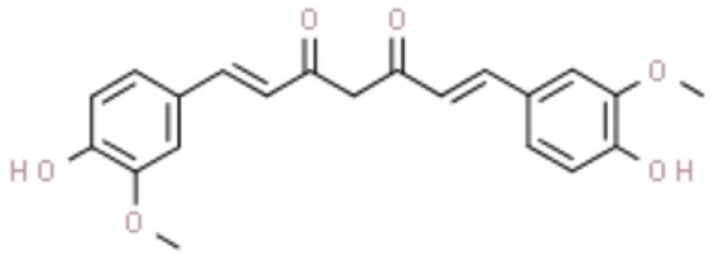
Structure of curcumin. It was drawn using ChemSpider, an online free chemical structure database.

Multiple epidemiological studies have explored the role of natural compounds in the development, progress, and survival of cancer cells [7]. Several natural compounds known for their antioxidant and chemotherapeutic properties, including soy, curcumin, resveratrol, and retinoids, have been reported as possible therapeutic compounds against glioblastoma [8]. Curcumin is a polyphenol extracted from the rhizome of the plant Curcuma longa, which belongs to the *Zingiberaceae* family. Curcumin has exhibited a prominent role in the treatment of several health conditions, including metabolic syndrome, inflammatory disorders, neurodegenerative diseases, as well as different types of cancer [9,10,11]. The majority of the antitumor effects of curcumin include cell-cycle arrest, inhibition of oncogenes, and increased apoptosis of cancer cells [12]. Curcumin’s antiproliferative effects are related to different molecular pathways, such as nuclear factor κB (NF-κB), Akt, and Wnt/β-catenin [13,14,15]. NF-κB is overexpressed in GBM and its deregulation is related to increased tumor growth and cell cycle progression, whereas the Wnt/β-catenin pathway affects cell proliferation, differentiation, and tumorigenesis [16,17]. Curcumin has been found to suppress the NF-κB signaling pathway through the blockage of constitutive Akt and JNK activation [18]. Moreover, through inhibition of the WNT/β-catenin pathway, curcumin can decrease the expression of cyclin D1 and thus inhibit the development and proliferation of gliomas [19]. The utilization of curcumin is associated with certain limitations, including poor oral bioavailability, rapid metabolism, and elimination. However, the use of nanotechnology drug-delivery systems such as liposomes, nanoparticles, or micelles can help overcome those limitations [20]. Furthermore, the molecular weight of curcumin is 368.38 Daltons and is thus able to cross the blood–brain barrier (BBB) (Figure 1).

Since radiotherapy belongs to the standard treatment of glioblastoma (GBM) patients, the radiosensitizing potential of curcumin in human glioma cells is worth taking note of. Both the dose range and timing of curcumin administration when combined with irradiation are important. The present study was designed to investigate the antitumor effects of curcumin, both alone and in combination with radiotherapy in glioblastoma cell lines. Our results suggest that the co-treatment of curcumin and radiation resulted in significant cell death and inhibited cell growth more effectively than either single treatment did.

## 2. Results

### 2.1. Curcumin Inhibits Glioblastoma Cell Proliferation

**Figure 2:**
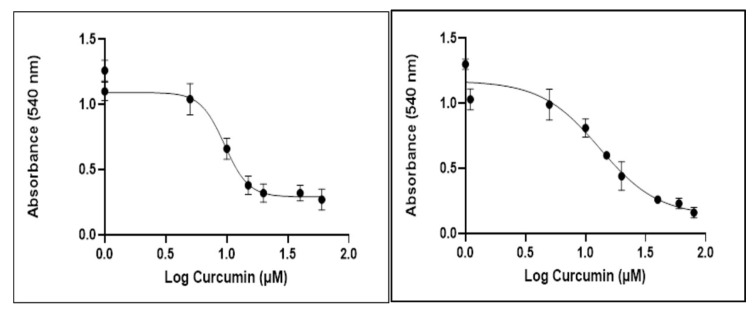
Cytotoxic effect of curcumin on glioblastoma cell lines U87 and T98 at 72 h. Values shown are the means and standard deviations from three independent experiments and are normalized to non-treated cells (*p* < 0.05 vs. control). The IC50 values were determined using the non-linear regression analysis model of GraphPad Prism Version 8.

**Figure 3:**
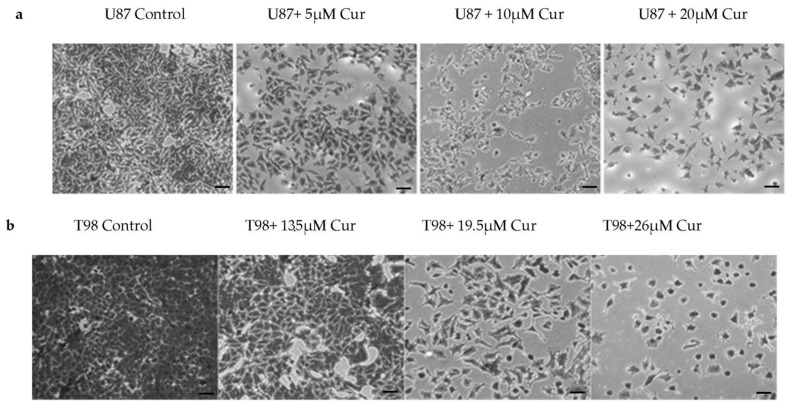
Morphological changes in U87 (**a**) and T98 (**b**) cell populations after treatment with crystal violet staining (0.2% Crystal Violet) (Scale bars = 50 μm). Images were recorded at 10× magnification. Cells were seeded in 6-well plates and 24 h later exposed to increasing curcumin concentrations. Crystal violet solution was added 48 h later and the cells were incubated at room temperature for 2–3 min. The excess crystal violet was removed, and plates were washed twice and left overnight to dry.

The effects of curcumin on cell viability are displayed in Figure 2. The IC50 value of curcumin determined after 72 h post-treatment was 10 μΜ in U87 cells and 13 μΜ in T98. To further delineate the effects of curcumin on cell proliferation, Crystal Violet staining of U87 and T98 cells was performed and photos using phase-contrast microscopy were taken at 72 h. Increasing concentrations of curcumin induced changes in the morphology of both cell lines, including cell shrinkage, indicating cell death (Figure 3a,b).

### 2.2. Combinatorial Effect of Curcumin and Irradiation on Glioblastoma Cells

Curcumin was used in concentrations ranging from 5–25 μΜ and 3.25–26 μΜ in U87 and T98 cells, respectively, whereas radiation was given in doses of 2 or 4 Gy. The effect of curcumin in combination with radiation is summarized in Table 1 and Table 2. In U87 cells, curcumin and radiation exerted synergism in the majority of tested combinations, and the highest synergy was monitored when curcumin was given at its IC50 value, namely 10 μΜ. In T98 cells, the highest levels of synergy were observed at higher curcumin concentrations, particularly at 26 μΜ, possibly due to the resistance of those cells to both chemotherapy and radiotherapy. Overall, curcumin and radiation exhibited a strong synergistic relationship in both cell lines, except for some mild antagonistic behavior at lower curcumin concentrations, 5 μΜ in U87 cells and 6.5 and 13 μΜ in T98 cells, possibly due to the inability of curcumin to sensitize glioblastoma cells to the cytotoxic effects of irradiation at lower doses; however, further studies are needed.

**Figure 4:**
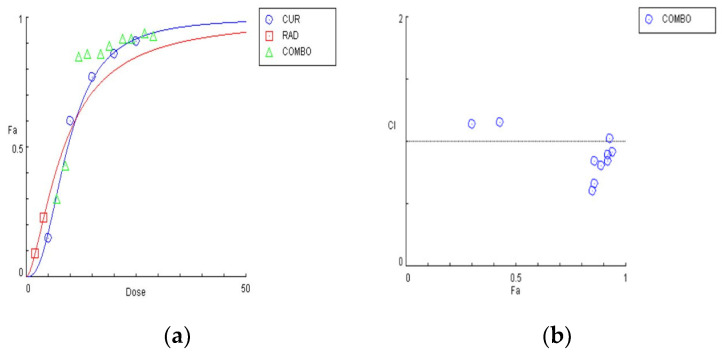
Graphical presentations obtained from the CompuSyn Report for the curcumin and radiation combination in U87 cells. (**a**) Dose–effect curve; (**b**) combination index plot.

**Figure 5:**
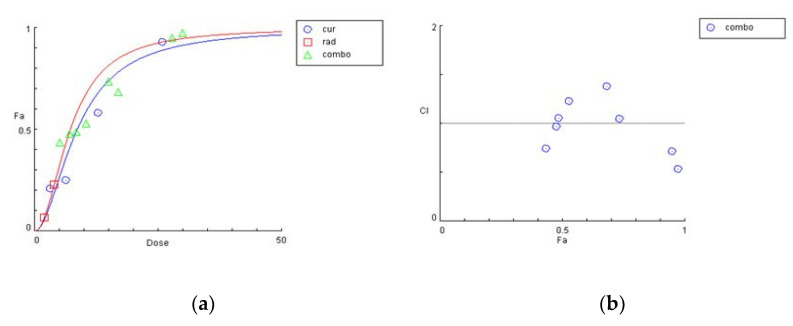
Graphical presentations obtained from the CompuSyn Report for the curcumin and radiation combination in T98 cells. (**a**) Dose–effect curve; (**b**) combination index plot.

The graphical representation of the combinatorial effect of curcumin and radiation is also presented via the dose–effect curves and combination index plots that were created using CompuSyn software for U87 (Figure 4a,b) and T98 (Figure 5a,b) cell lines.

**Figure 6:**
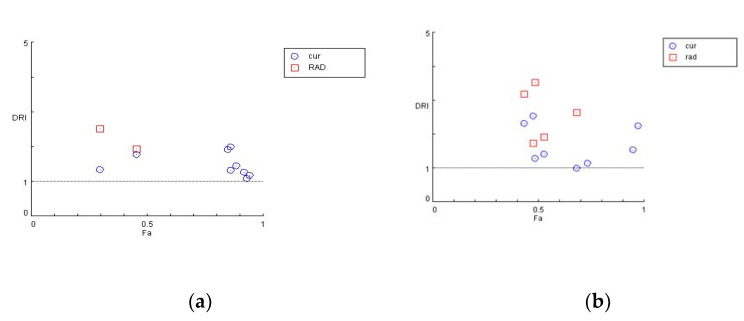
Dose reduction plots for the combination of curcumin and radiation at different experimental points for U87 (**a**) and T98 (**b**) cells. DRI >1 shows favorable dose reduction of both factors.

For each drug combination, CompuSyn calculates the dose–reduction index where DRI >1 and < 1 indicate a favorable and not favorable dose–reduction, respectively, and DRI = 1 indicates no dose-reduction. As seen in Figure 6, in both U87 (a) and T98 (b) cell lines, most combinations of curcumin and radiation show a favorable dose–reduction (DRI > 1).

### 2.3. Curcumin Enhanced the Radiation-Induced G2/M Arrest in Glioblastoma Cells

**Figure 7:**
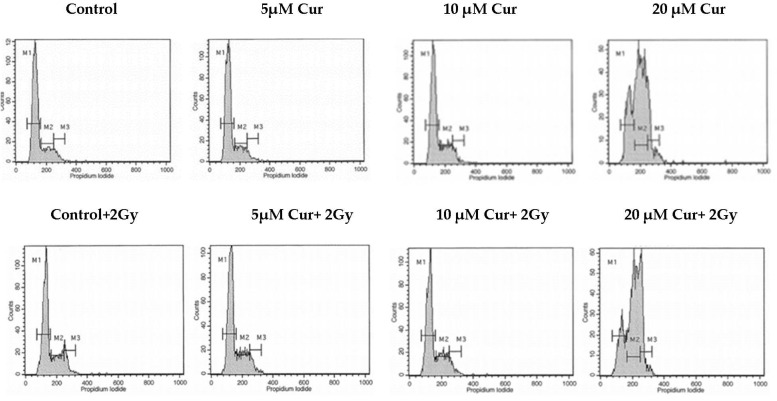
Histogram representation of cell-cycle distribution in U87 cells after treatment with increasing curcumin concentrations. Cells (10^4^) were seeded in 24-well plates and after 24 h were exposed to different curcumin concentrations; 2 h later, the plates were irradiated with 2 Gy. After 72 h, the cells were stained with propidium iodide and the DNA content was observed.

**Figure 8:**
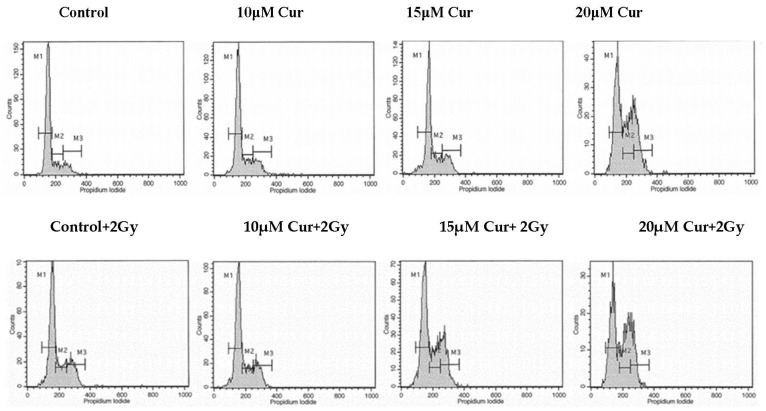
Histogram representation of cell-cycle distribution in T98 cells after treatment with increasing curcumin concentrations. Following the same procedure, the cells were stained with propidium iodide at 72 h, and the DNA content was observed. Curcumin and radiation co-treatment induced G2/M arrest in glioblastoma cells.

Since curcumin treatment in combination with radiation brought significant cytotoxicity in both U87 and T98 cell lines, it was important to explore how the combination treatment affected different phases of cell-cycle progression. For this, a flow cytometric analysis with DNA staining dye, propidium iodide (PI), was performed. Cell cultures were treated with increasing curcumin concentrations for 72 h. At 72 h, the cells were stained with PI and the DNA content was calculated. Curcumin induced both an S and G2/M arrest in U87 and T98 cells when given alone. Interestingly, when cells were treated with IC50 and 2IC50 concentrations of curcumin along with 2 Gy of radiation, the percentage distribution of the cells in the G2/M phase was enhanced considerably in both U87 (Figure 7) and T98 (Figure 8) cell lines. Specifically, when U87 cells were treated with 20 μΜ of curcumin, followed by 2 Gy, the percentage distribution of cells in the G2/M phase (20.9%) was enhanced significantly compared to treatment with curcumin alone (13%), under the same conditions. Accordingly, in the T98 cell line, treatment with 15 μΜ of curcumin, followed by 2 Gy, resulted in a higher-percentage distribution of cells in the G2/M phase (22.9%) compared to treatment with 15 μΜ of curcumin alone (13.5%). Since the G2/M phase is arrested as a result of DNA damage, the above results show that curcumin may be enhancing the damaging effects of radiation.

### 2.4. Curcumin and Temozolomide Exhibited Synergistic Anti-Proliferative Effect on Glioma Cells

**Figure 9:**
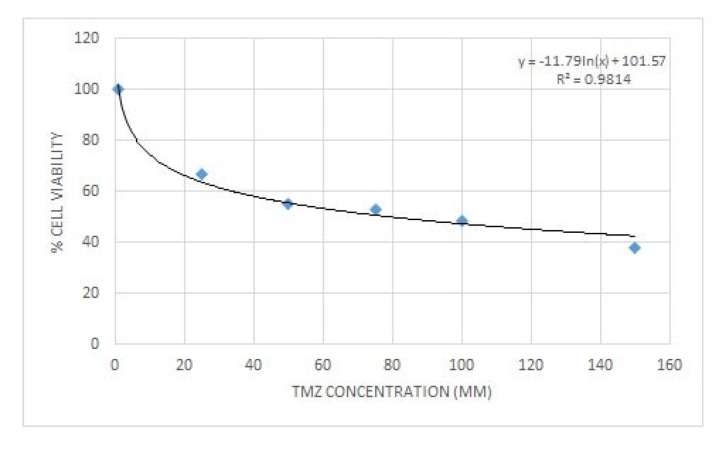
Cytotoxic effect of temozolomide on glioblastoma cell line U87. The IC50 value for temozolomide was calculated at 80 μΜ with an R^2^ = 0.9814.

Prior to examining the synergistic effect of curcumin and ΤΜΖ, the effective dose for ΤΜΖ was determined using the trypan blue exclusion assay. U87 cells were seeded at a density of 10^4^ in 24-well plates for 24 h and then increased concentrations of ΤΜΖ were added. The cells were incubated for another 72 h, at the end of which trypan blue dye was added to each well. The cytotoxic effects of ΤΜΖ in U87 cells are presented in Figure 9.

**Figure 10:**
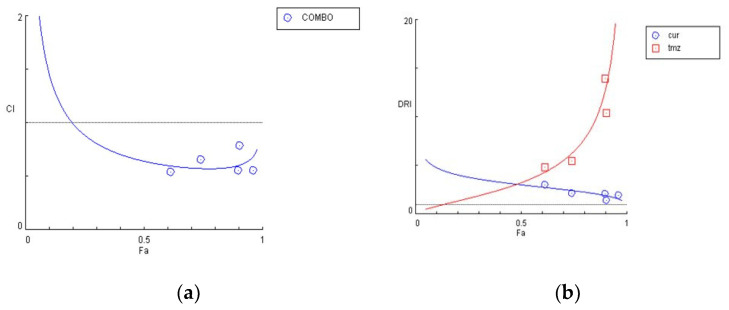
Graphical presentations obtained from the CompuSyn Report for the curcumin and temozolomide combination in U87 cells. (**a**) Combination index plot; (**b**) dose–reduction plot.

To determine the synergism or antagonism of the combination of curcumin and ΤΜΖ in U87 cells, curcumin was used in concentrations ranging from 2.5–20 μΜ in U87, whereas TMZ was given in concentrations ranging 20–160 μΜ. Combination therapy was carried out in a constant ratio of 1:8 (cur: ΤΜΖ) and the combined effect of the two drugs factors was compared with the effect of each drug separately. Curcumin and ΤΜΖ exerted strong synergism in all combinations tested (CI < 1). When given together, a favorable dose–reduction effect was observed for both drugs on each combination point (DRI > 1). Both combination index and dose–reduction plots were developed with the use of CompuSyn (Figure 10a,b).

### 2.5. Zebrafish Lethal Concentration Determination

**Figure 11:**
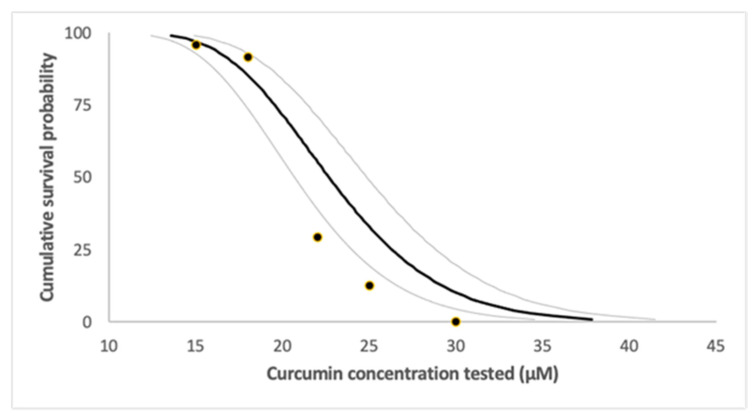
Lethal concentration determination.

The toxic effect is induced in a concentration-dependent manner. The mortality rate rises as the concentration increases. The lethal dose was LC50 = 20.89 μΜ, while LC25 and LC75 were 18.50 μΜ and 23.35 μM, respectively (Figure 11).

## 3. Discussion

Glioblastoma is the most common and severe tumor of the CNS [1]. Many chemotherapeutic agents have been used against glioblastoma, including TMZ [3]. Radiotherapy and chemotherapy belong to the standard treatment of glioblastoma, following surgical resection. However, both chemo and radio resistance has been proven to be a major challenge for the successful treatment of GBM. Researchers have attempted to identify novel radiosensitizers to achieve better clinical outcomes, including natural compounds.

Curcumin is a natural polyphenol that has been used for centuries in traditional Chinese medicine in the treatment of allergies, infections, and respiratory disorders [21]. In recent years, phytochemicals, and curcumin in particular, have garnered the interest of various scientific groups worldwide in both experimental and pre-clinical studies for their effects on different types of cancer.

The present study unveiled that curcumin and radiation are an effective combination in the treatment of glioblastoma in vitro. Curcumin inhibited cell proliferation and caused cell-cycle arrest in both U87 and T98 cell lines. When combined with irradiation, its anti-proliferative properties were further enhanced. In a zebrafish model, no significant mortality was observed at curcumin concentrations of up to 18 μΜ. Since glioblastoma is difficult to cure via neurosurgery or radiotherapy alone, the combination of curcumin and radiation could be a potentially promising treatment.

In previous studies, curcumin was investigated for its ability to exert radiosensitizing effects on different cell types. Kunwar et al. examined the cellular uptake of curcumin and confirmed that cancer cells (T cell lymphoma of murine origin and human breast cancer cells) showed relatively higher uptake of curcumin compared to normal cells (mouse spleen lymphocytes and fibroblast cells) [22]. Zanotto-Filho et al. studied the effects of curcumin on the proliferation of glioblastoma cell lines in vitro, as well as in a preclinical model in vivo. Curcumin induced cell death and inhibited proliferation in four glioma cell lines at IC50 values of 19–28 μΜ. In vivo, curcumin reduced the size of intracranially growing tumors in rats, and showed no evidence of toxicity in healthy tissues [23]. Rodriguez et al. investigated the role of curcumin in glioblastoma, analyzing 19 in vitro and 5 in vivo studies. All studies showed that curcumin induced cell death through a series of molecular mechanisms, including the activation of apoptotic pathways via caspace-3, p21, and p53 [24].

Glioblastoma remains the most aggressive and invasive primary brain tumor in adults. Current treatment includes surgical excision, followed by chemotherapy and radiotherapy. However, despite aggressive treatment, recurrence is, in most cases, an inevitable event [2]. The presence of BBB is an important contributor to glioblastoma’s resistance to chemotherapy. Curcumin, theoretically, can pass through the BBB thanks to its relatively low molecular weight and lipophilic nature. [10] In most of the studies carried out on animals, curcumin was given orally [25]. In rats, after oral administration of a 2 g/kg dose, curcumin reached a maximum serum concentration of 1.35 μg/mL. However, when the same dosage was given to humans, the serum levels of curcumin were extremely low (0.006 μg/mL). When mice were given 50 mg/kg curcumin orally, a brain concentration lower than the limit of detection was observed 60 or 120 min after administration [26]. On the contrary, when mice were fed for up to 4 months with curcumin (2.5–10 mg/day orally), they showed 0.5 µg/g brain tissue [27]. The low absorption rate, its rapid metabolism, and systemic elimination are the major obstacles that deprive it from reaching satisfactory serum levels in humans after oral administration [28].

Radiotherapy remains a significant part of every treatment regimen against glioblastoma; however, it has not been used thoroughly in the investigation of combination treatments for GBM [29]. The possible photosensitizing effects of curcumin in vitro have been explored by a few scientific groups. Jamali et al. examined the effects of curcumin on the DKMG human cell line when given along with photodynamic therapy (radiation dose 60 J/cm^2^). Their results indicated an important curcumin dose–reduction in cell proliferation [30]. Back in 2007, Dhandapani et al. investigated the combinatorial effects of curcumin and irradiation. In their study, they treated two glioma lines, U87 and T98, with curcumin alone at a concentration of 25 μΜ, irradiation alone (5 Gy using a 137Cs γ-cell-40 Exactor), and their combination. They observed that a synergistic effect of curcumin and radiation existed in both cell lines as a result of the inhibition of anti-apoptotic gene expression and that the maximum% cell death induced by this combination was about 60% [18]. In the present study, we found that the combination of curcumin and radiation can induce cytotoxicity of up to 94% and 95% for the U87 and T98 cell lines, respectively, accompanied by favorable dose–reduction. For glioblastoma, based on the pivotal phase 3 trial published in 2005, standard treatment includes 60 Gy in 2 Gy fractions delivered over 6 weeks [31]. Taking into account our previous studies as well, and in order to follow the standard treatment protocol, we used a dose of 2 or 4 Gy using X-rays generated by a linac 6 MV accelerator [32]. The most prominent synergistic effect was observed when curcumin was given at its IC50 value, namely 10 μΜ for the U87 cell line, whereas for the more-resistant T98 cell line, a concentration of 26 μΜ demonstrated the highest synergy. In both cases, 2 Gy of radiation was enough to produce that strong synergetic effect.

Curcumin induced G2/M cell-cycle arrest when given alone and that effect was further enhanced when combined with irradiation. Back in 2015, Zhang et al. investigated the potent radiosensitizing effects of curcumin on U87 human glioma cells in vivo. Nude mice bearing subcutaneous U87 xenografts were treated with 50 mg/kg of curcumin and exposed to 5 Gy of irradiation. The results showed that curcumin significantly increased the radiosensitivity of U87 cells in vivo via the enhancement of the dual-specificity phosphatase (DUSP-2) pathway [33]. The exact molecular mechanisms of the radiosensitization of curcumin are still under investigation; however, it is known that drugs that produce G2/M arrest are potent radiosensitizers [34]. When DNA damage occurs, the DNA damage checkpoint is activated, which involves ATM kinase activation and autophosphorylation at Ser1981. This can induce cell-cycle arrest to delay the proliferation of cancer cells. Since cells in the G2/M phase are more sensitive to radiation, this arrest may be a significant strategy in the treatment of GBM [35]. In the present study, we found that low doses of curcumin increased the number of cells undergoing G2/M phase arrest. Radiation can also result in G2/M phase arrest and apoptosis. Thus, the addition of curcumin can induce the transformation of cancer cells into a more radiosensitive status. When curcumin was given with ΤΜΖ, which is the major chemotherapeutic drug against glioblastoma, a synergistic anti-proliferative effect on both cell lines was also observed. Therefore, a combinatorial treatment using curcumin, temozolomide, and low doses of irradiation may be a promising future treatment option. Preclinical studies on the combinatorial effect of curcumin and radiation are depicted in Table 3. Our current results show that a combinatorial treatment using curcumin and low doses of irradiation may be a promising future treatment option.

The present study has several limitations. Following repeated intake of curcumin in humans, plasma concentrations have been found to be relatively low, peaking at approximately 2 μΜ [37]. Mean intratumoral concentrations of curcumin have been reported to be around 0.15 μΜ after oral administration [38]. Novel technical approaches to increase the bioavailability of curcumin include encapsulation in nanoparticles, the use of liquid micelles, or micronized powder [39,40]. Moreover, evaluating the combined effects of curcumin and radiation 72 h after treatment may be preliminary and may require additional experiments, including a colony-forming assay where the effects on cell viability are more prominently observed 10–14 days after treatment [41]. Therefore, further experiments are needed to fully determine the degree of synergy between curcumin and radiation in glioblastoma cells. Although we found synergistic anti-cancer effects of curcumin and radiation in cultured glioblastoma cells lines, the mechanistic details of glioblastoma growth, proliferation, invasion, and metastasis in animal or human brains are much more complex. For this reason, a complete understanding of the mechanism of the combined effects of curcumin and radiotherapy will require additional experiments in animals to optimize the therapeutic strategy prior to clinical use.

In summary, this study is the first to demonstrate that co-treatment of curcumin and radiation shows higher inhibitory effects compared to their individual administration and results in a more prominent G2/M arrest in the cell cycle of both U87 and T98 cell lines. Given that glioblastoma is a highly heterogenic tumor, difficult to treat, with the additional obstacle of the presence of BBB, the need for novel and effective anti-cancer drugs is a clearly unmet clinical need. Further studies will be necessary to better understand the synergistic effects of curcumin and radiation on glioblastoma treatment and validate our results in glioma xenograft models prior to clinical trials.

## 4. Materials and Methods

### 4.1. Cell Lines and Treatment Conditions

The human glioma cell line T98 was obtained from ATCC (Manassas, VA, USA), whereas the U87 cell line was obtained from Dr W.K. Alfred Yung (Department of Neuro-Oncology, M.D. Anderson Cancer Center, Houston, TX, USA). Both cell lines were cultured in Dulbecco’s Modified Eagle’s Medium (Gibco BRL, Life Technologies, Grand Island, NY, USA), supplemented with 10% fetal bovine serum (FBS) and 1% penicillin–streptomycin (Gibco BRL). Both cell lines were incubated in a humidified atmosphere regulated at 5% CO_2_ and 37 °C. The medium was changed every three days. Curcumin (Sigma Aldrich, St. Louis, MO, USA) was dissolved in DMSO to a stock concentration of 36 mM and stored at 4 °C. TMZ (Sigma Aldrich, St. Louis, MO, USA) was dissolved in dimethyl sulfoxide and stored as a stock solution of 103 mM at 4 °C. Before each experiment, curcumin and TMZ were diluted from their stock solution to the final concentration with a culture medium. Less than 1% of DMSO was present in the final volumes of each experiment. Cultures of glioma cells were treated with curcumin alone or in combination with radiotherapy or TMZ.

### 4.2. Viability Assay

Cultures of human glioma cells were treated with curcumin in concentrations of 1, 5, 10, 15, 20, 40, and 60 µM for the U87 cell line and in concentrations of 1, 5, 10, 15, 20, 40, 60, and 80 µM for the T98 cell line. Cell viability was evaluated by the Trypan Blue exclusion assay and the 3-(4,5-dimethylthiazol-2-yl)-2,5-diphenyltetrazolium bromide (MTT, Sigma Aldrich, St. Louis, MO, USA assay) [42,43]. A Trypan Blue exclusion assay was performed in 24-well plates where 10,000 cells were seeded, and after 24 h, were exposed to increasing concentrations of curcumin. Cell viability was determined at 72 h with the use of a phase-contrast microscope. For the MTT assay, 2000 cells were seeded in 96-well plates, and after 24 h, were exposed to the same increasing concentrations of curcumin. The cells were incubated for another 72 h and then MTT was added. The amount of MTT-formazan was determined at 570 nm. Both methods were performed three times, and the results presented are the mean of the three. Changes in cell proliferation were also continuously determined with the use of a phase-contrast microscope. The Trypan Blue assay was also used for the determination of cell viability in U87 cells after exposure to increased concentrations of TMZ.

### 4.3. Crystal Violet Assay

The crystal violet assay was used to further determine cell proliferation in both U87 and T98 cell lines after exposure to increased curcumin concentrations. Cells were seeded at a density of 105 per well in 6-well plates, and after 24 h, curcumin was added in increased concentrations. The cells were incubated for 48 h, washed twice with phosphate-buffered saline (PBS), and further incubated for 2–3 min with the Crystal Violet Solution 0.2% (0.2 g Crystal Violet Powder, MERCK, MA, USA) in 80 mL of ddH_2_O and 20 mL Methanol. Plates were then rinsed with running water and left overnight to dry. Pictures of each well-plate were taken the next day with the use of phase-contrast microscopy.

### 4.4. Flow Cytometric Analysis of DNA Cell Cycle

Cells (10^4^) were treated with increased concentrations of curcumin alone or in combination with radiation. Untreated cells were used as a negative control with 1% of DMSO. At least three independent experiments were performed, and all samples were run in triplicates. Flow cytometric analysis was performed on day 5. For the DNA cell cycle, cells were treated with trypsin, centrifuged, washed well with PBS, and then incubated with PI-working solution (50 µg/mL PI, 20 mg/mL RNase A, and 0.1% Triton X-100) for 20 min at 37 °C in the dark. With the use of a flow cytometer (FACScalibur, BD Biosciences, San Jose, CA, USA), the PI fluorescence of 10,000 individual nuclei was determined. Using the CellQuest software program (BD Biosciences, CA, USA), the fractions of cells in G0/G1, S, G2/M, and sub-G0/G1 phases were analyzed [44].

### 4.5. Combination Treatment with Curcumin and Radiation

Cells (10^4^) were treated with different concentrations of either curcumin alone or a combination of curcumin and radiation. U87 and T98 cells were cultured in 24-well plates and after 24 h were treated with curcumin. After 2 h, the cells were irradiated at 2 Gy or 4 Gy as described previously [32]. Cell viability was determined using the Trypan Blue exclusion assay at 72 h. The combinatorial effect of curcumin and radiation was evaluated using the combination index method of Chou and Talalay [45]. Curcumin was used in concentrations of 5, 10, 15, 20, and 25 µM for the U87 cell line and at concentrations of 3.25, 6.5, 13, and 26 µM for the T98 cell line. Two different doses of irradiation were used in both cell lines, 2 and 4 Gy. A total of 10 and 8 different combinations with three replicates per condition were used for the U87 and T98 cell lines, respectively. The affected fraction of cells after treatment with curcumin alone, irradiation alone, or different combinations of those two was calculated, and the dose–effect curves were generated. The Combination Index (CI) was determined using CompuSyn software (Compusyn, Inc., Paramus, NJ, USA). The CI value determines the effect of the combination treatment. A CI < 1 is considered synergistic, a CI = 1 is considered additive, and a CI > 1 is considered antagonistic [46].

### 4.6. Combination Treatment with Curcumin and Temozolomide

Cells (10^4^) were treated with different concentrations of either curcumin, TMZ, or a combination of curcumin and TMZ. U87 cells were cultured in 24-well plates and after 24 h were treated with curcumin and/or TMZ, and the Trypan Blue exclusion assay was performed at 72 h. The dose–effect parameters of each drug alone or in different combinations were automatically determined from the median-effect equation created by CompuSyn software.

### 4.7. Zebrafish

#### 4.7.1. Zebrafish Housing and Husbandry

Adult zebrafish of the wild-type strain (AB) were maintained in a colony room in a recirculated system at 28 ± 1 °C, pH 6.5–7.5, conductivity 500 ± 50 μS cm^−1^ with a 14-h light/10-h dark photoperiod (lights on at 8:00 a.m.). Feeding of the fish was performed twice per day following common practices (with zebrafish feed). Sexually mature zebrafish (at least three-months old) were used for spawning. Embryos were collected and pooled into a standard zebrafish E3 culture medium (5 mmol/L NaCl, 0.33 mmol/L CaCl_2_, 0.33 mmol/L MgSO_4_·7H_2_O, and 0.17 mmol/L KCl).

#### 4.7.2. Zebrafish Toxicity Testing

The collection of zebrafish embryos was performed at the beginning of the 14 h light cycle following the mating procedure that took place overnight. After the inspection of the embryos, those that were unfertilized or showed significant malformation were removed, and the dechorionation process followed at 24 hpf. The dechorionated embryos were placed in 24-well culture plates (2 embryos per well, 1.5 mL of solution per well) and each experiment was performed in triplicate. In the current study, five different concentrations of curcumin were tested (0, 15, 18, 22, 25, 30 μΜ). In total, 248 embryos were studied, of which 48 per each concentration were in the control group.

#### 4.7.3. Lethal Concentration (LC50) Determination

Preliminary tests were performed in order to evaluate the full 0–100% range of mortality. The concentration range was 15 to 30 μΜ. Toxicity assays (LC50 calculation) and confidence intervals (LC25 and LC75) were determined based on cumulative mortality at the end of the experiment.

### 4.8. Statistical Analysis

All results are presented as the mean ± standard deviation (SD). IC50 values were determined with the use of GraphPad Prism software (v. 8.0.0, San Diego, California USA, Trial Version) through regression analysis. Multiple comparisons of groups were analyzed using two-way ANOVA with the post hoc Tukey test. Parameters of LC50 were assessed using a regression Probit analysis (the chi-square test, Pearson goodness of fit test, and 95% confidence interval). Analyses were performed using SPSS statistical software v26 (IBM Corp., Armonk, NY, USA). Differences were considered significant at *p*-values < 0.05.

## Figures and Tables

**Table 1:** Assessment of combinatorial effect of curcumin and radiation in U87 cells. CI was determined by CompuSyn software.

Curcumin (μM)	Radiation (Gy)	Effect	CI	Conclusion
5	2	0.3	1.14445	Antagonism
10	2	0.85	0.6062	Synergism
15	2	0.86	0.84063	Synergism
20	2	0.92	0.84358	Synergism
25	2	0.94	0.91683	Synergism
5	4	0.43	1.15321	Antagonism
10	4	0.86	0.66052	Synergism
15	4	0.89	0.81149	Synergism
20	4	0.92	0.89425	Synergism
25	4	0.93	1.02773	Antagonism

**Table 2:** Assessment of combinatorial effect of curcumin and radiation in T98 cells. CI was determined by CompuSyn software.

Curcumin (μM)	Radiation (Gy)	Effect	CI	Conclusion
3.25	2	0.44	0.74507	Synergism
6.5	2	0.49	1.0575	Antagonism
13	2	0.74	1.04795	Antagonism
26	2	0.95	0.71409	Synergism
3.25	4	0.48	0.97007	Synergism
6.5	4	0.53	1.22819	Antagonism
13	4	0.68	1.38199	Antagonism
26	4	0.97	0.53544	Synergism

**Table 3:** Studies on the combinatorial effect of curcumin and irradiation in different in vitro and in vivo GBM models.

In Vitro/In Vivo Effect	Mechanism of Action	Dosing/Duration	References
Synergetic effect of curcumin when combined with irradiation on T98G and U87MG cells in vitro	Decrease in anti-apoptotic gene expression	25 μM curcumin 6 h prior to 5 Gy radiation	Dhandapani et al. [18]
In vivo radiosensitization of U87 glioma xenografts in vivo	Upregulation of DUSP-2, inhibition of ERK/JNK phosphorylation	50 mg/kg plus irradiation (5 Gy) every 2 days, curcumin 2 h prior to radiation	Zhang et al. [33]
No radiosensitizing effect of curcumin on cell viability in U251 glioma cells in vitro	Clonogenic cell survival in U251 cells is reduced after 96 h at doses exceeding 5 μM	5 μM curcumin 72 h prior to 1–6 Gy single dose	Sminia et al. [36]

## References

[1] Klinger N.V., Mittal S. (2016). Therapeutic Potential of Curcumin for the Treatment of Brain Tumors. Oxid. Med. Cell Longev..

[2] Weathers S.P., Gilbert M.R. (2014). Advances in treating glioblastoma. Prime Rep..

[3] Tsamis K.I., Alexiou G.A., Vartholomatos E., Kyritsis A.P. (2013). Combination treatment for glioblastoma cells with tumor necrosis factor-related apoptosis-inducing ligand and oncolytic adenovirus delta-24. Cancer Investig..

[4] Kyritsis A.P., Levin V.A. (2011). An algorithm for chemotherapy treatment of recurrent glioma patients after temozolomide failure in the general oncology setting. Cancer Chemother. Pharmacol..

[5] Alexiou G.A., Goussia A., Voulgaris S., Fotopoulos A.D., Fotakopoulos G., Ntoulia A., Zikou A., Tsekeris P., Argyropoulou M.I., Kyritsis A.P. (2012). Prognostic significance of MRP5 immunohistochemical expression in glioblastoma. Cancer Chemother. Pharmacol..

[6] Sandberg C.J., Altschuler G., Jeong J., Strømme K.K., Stangeland B., Murrell W., Grasmo-Wendler U.H., Myklebost O., Helseth E., Vik-Mo E.O. (2013). Comparison of glioma stem cells to neural stem cells from the adult human brain identifies dysregulated Wnt- signaling and a fingerprint associated with clinical outcome. Exp. Cell Res..

[7] Sarkar F.H., Li Y., Wang Z., Kong D. (2009). Cellular signaling perturbation by natural products. Cell. Signal..

[8] Kyritsis A.P., Bondy M.L., Levin V.A. (2011). Modulation of glioma risk and progression by dietary nutrients and antiinflammatory agents. Nutr. Cancer..

[9] Yung W.K., Kyritsis A.P., Gleason M.J., Levin V.A. (1996). Treatment of recurrent malignant gliomas with highdose 13-cis-retinoic acid. Clin. Cancer Res..

[10] Unlu A., Nayir E., Kalenderoglu M.D., Kirca O., Ozdogan M. (2016). Curcumin (Turmeric) and cancer. J. BUON.

[11] Hewlings S.J., Kalman D.S. (2017). Curcumin: A Review of Its’ Effects on Human Health. Foods.

[12] Zoi V., Galani V., Lianos G.D., Voulgaris S., Kyritsis A.P., Alexiou G.A. (2021). The Role of Curcumin in Cancer Treatment. Biomedicines.

[13] Mukhopadhyay A., Banerjee S., Stafford L.J., Xia C., Liu M., Aggarwal B.B. (2002). Curcumin-induced suppression of cell proliferation correlates with down-regulation of cyclin D1 expression and CDK4-mediated retinoblastoma protein phosphorylation. Oncogene.

[14] Hatanpaa K.J., Burma S., Zhao D., Habib A.A. (2010). Epidermal growth factor receptor in glioma: Signal transduction, neuropathology, imaging, and radioresistance. Neoplasia.

[15] Elamin M.H., Shinwari Z., Hendrayani S.F., Al-Hindi H., Al-Shail E., Khafaga Y., Aboussekhra A. (2010). Curcumin inhibits the Sonic Hedgehog signaling pathway and triggers apoptosis in medulloblastoma cells. Mol. Carcinog..

[16] Nagai S., Washiyama K., Kurimoto M., Takaku A., Endo S., Kumanishi T. (2002). Aberrant nuclear factor-κB activity and its participation in the growth of human malignant astrocytoma. J. Neurosurg..

[17] Bahrami A., Amerizadeh F., ShahidSales S., Khazaei M., Ghayour-Mobarhan M., Sadeghnia H.R., Avan A. (2017). Therapeutic potential of targeting Wnt/β-catenin pathway in treatment of colorectal cancer: Rational and progress. J. Cell. Biochem..

[18] Dhandapani K.M., Mahesh V.B., Brann D.W. (2007). Curcumin suppresses growth and chemoresistance of human glioblastoma cells via AP-1 and NFκB transcription factors. J. Neurochem..

[19] He M., Li Y., Zhang L., Li L., Shen Y., Lin L., Zheng W., Chen L., Bian X., Ng H.K. (2014). Curcumin suppresses cell proliferation through inhibition of the Wnt/β-catenin signaling pathway in medulloblastoma. Oncol. Rep..

[20] Mirzaei H., Khoi M.J.M., Azizi M., Goodarzi M. (2016). Can curcumin and its analogs be a new treatment option in cancer therapy?. Cancer Gene Ther..

[21] Ammon H.P., Wahl M.A. (1991). Pharmacology of Curcuma longa. Planta Med..

[22] Kunwar A., Barik A., Mishra B., Rathinasamy K., Pandey R., Priyadarsini K.I. (2008). Quantitative cellular uptake, localization and cytotoxicity of curcumin in normal and tumor cells. Biochim. Biophys. Acta Gen. Subj..

[23] Zanotto-Filho A., Braganhol E., Edelweiss M.I., Behr G.A., Zanin R., Schröder R., Simões-Pires A., Battastini A.M., Moreira J.C. (2012). The curry spice curcumin selectively inhibits cancer cells growth in vitro and in preclinical model of glioblastoma. J. Nutr. Biochem..

[24] Rodriguez G.A., Shah A.H., Gersey Z.C., Shah S.S., Bregy A., Komotar R.J., Graham R.M. (2016). Investigating the therapeutic role and molecular biology of curcumin as a treatment for glioblastoma. Ther. Adv. Med. Oncol..

[25] Prasad S., Tyagi A.K., Aggarwal B.B. (2014). Recent developments in delivery, bioavailability, absorption and metabolism of curcumin: The golden pigment from golden spice. Cancer Res. Treat..

[26] Schiborr C., Eckert G.P., Rimbach G., Frank J. (2010). A validated method for the quantification of curcumin in plasma and brain tissue by fast narrow-bore high-performance liquid chromatography with fluorescence detection. Anal. Bioanal. Chem..

[27] Begum A.N., Jones M.R., Lim G.P., Morihara T., Kim P., Heath D.D., Rock C.L., Pruitt M.A., Yang F., Hudspeth B. (2008). Curcumin structure-function, bioavailability, and efficacy in models of neuroinflammation and Alzheimer’s disease. J. Pharmacol. Exp. Ther..

[28] Jamwal R. (2018). Bioavailable curcumin formulations: A review of pharmacokinetic studies in healthy volunteers. J. Integr. Med..

[29] Levin V., Maor M., Thall P., Yung W., Bruner J., Sawaya R., Kyritsis A., Leeds N., Woo S., Rodríguez L. (1995). Phase II study of accelerated fractionation radiation therapy with carboplatin followed by vincristine chemotherapy for the treatment of glioblastoma multiforme. Int. J. Radiat. Oncol. Biol. Phys..

[30] Jamali Z., Hejazi S.M., Ebrahimi S.M., Moradi-Sardareh H., Paknejad M. (2018). Effects of LED-Based photodynamic therapy using red and blue lights, with natural hydrophobic photosensitizers on human glioma cell line. Photodiagn. Photodyn. Ther..

[31] Stupp R., Mason W.P., van den Bent M.J., Weller M., Fisher B., Taphoorn M.J., Belanger K., Brandes A.A., Marosi C., Bogdahn U. (2005). Radiotherapy plus concomitant and adjuvant temozolomide for glioblastoma. N. Engl. J. Med..

[32] Alexiou G., Vartholomatos E., Tsamis K.I., Peponi E., Markopoulos G., Papathanasopoulou V., Tasiou I., Ragos V., Tsekeris P., Kyritsis A. (2019). Combination treatment for glioblastoma with temozolomide, DFMO and radiation. J. BUON.

[33] Zhang L., Ding X., Huang J., Jiang C., Cao B., Qian Y., Cheng C., Dai M., Guo X., Shao J. (2015). In vivo Radiosensitization of human glioma U87 cells induced by upregulated expression of DUSP-2 after treatment with curcumin. Curr. Signal Transd. Ther..

[34] Wang Y., Yang L., Zhang J., Zhou M., Shen L., Deng W., Liang L., Hu R., Yang W., Yao Y. (2018). Radiosensitization by irinotecan is attributed to G2/M phase arrest, followed by enhanced apoptosis, probably through the ATM/Chk/Cdc25C/Cdc2 pathway in p53-mutant colorectal cancer cells. Int. J. Oncol..

[35] Pawlik T.M., Keyomarsi K. (2004). Role of cell cycle in mediating sensitivity to radiotherapy. Int. J. Radiat. Oncol. Biol. Phys..

[36] Sminia P., van den Berg J., van Kootwijk A., Hageman E., Slotman B.J., Verbakel W. (2021). Experimental and clinical studies on radiation and curcumin in human glioma. J. Cancer Res. Clin. Oncol..

[37] Cheng A.L., Hsu C.H., Lin J.K., Hsu M.M., Ho Y.F., Shen T.S., Ko J.Y., Lin J.T., Lin B.R., Ming-Shiang W. (2001). Phase I clinical trial of curcumin, a chemopreventive agent, in patients with high-risk or pre-malignant lesions. Anticancer Res..

[38] Dutzmann S., Schiborr C., Kocher A., Pilatus U., Hattingen E., Weissenberger J., Gesler F., Quick-Weller J., Franz K., Seifert V. (2016). Intratumoral concentrations and effects of orally administered micellar Curcuminoids in Glioblastoma patients. Nutr. Cancer.

[39] Schiborr C., Kocher A., Behnam D., Jandasek J., Toelstede S., Frank J. (2014). The oral bioavailability of curcumin from micronized powder and liquid micelles is significantly increased in healthy humans and differs between sexes. Mol. Nutr. Food Res..

[40] Kanai M., Imaizumi A., Otsuka Y., Sasaki H., Hashiguchi M., Tsujiko K., Matsumoto S., Ishiguro H., Chiba T. (2012). Dose-escalation and pharmacokinetic study of nanoparticle curcumin, a potential anticancer agent with improved bioavailability, in healthy human volunteers. Cancer Chemother. Pharmacol..

[41] Franken N.A., Rodermond H.M., Stap J., Haveman J., van Bree C. (2006). Clonogenic assay of cells in vitro. Nat. Protoc..

[42] Kastamoulas M., Chondrogiannis G., Kanavaros P., Vartholomatos G., Bai M., Briasoulis E., Arvanitis D., Galani V. (2013). Cytokine effects on cell survival and death of A549 lung carcinoma cells. Cytokine.

[43] Alexiou G.A., Tsamis K.I., Vartholomatos E., Peponi E., Tzima E., Tasiou I., Lykoudis E., Tsekeris P., Kyritsis A.P. (2015). Combination treatment of TRAIL, DFMO and radiation for malignant glioma cells. J. Neuro-Oncol..

[44] Chondrogiannis G., Kastamoulas M., Kanavaros P., Vartholomatos G., Bai M., Baltogiannis D., Sofikitis N., Arvanitis D., Galani V. (2014). Cytokine Effects on Cell Viability and Death of Prostate Carcinoma Cells. BioMed Res. Int..

[45] Chou T.C. (2006). Theoretical Basis, Experimental Design, and Computerized Simulation of Synergism and Antagonism in Drug Combination Studies. Pharmacol. Rev..

[46] Chou T.C. (2010). Drug Combination Studies and Their Synergy Quantification Using the Chou-Talalay Method. Cancer Res..

